# Endosomal pH in neuronal signaling and synaptic transmission: role of Na^+^/H^+^ exchanger NHE5

**DOI:** 10.3389/fphys.2013.00412

**Published:** 2014-01-13

**Authors:** Graham H. Diering, Masayuki Numata

**Affiliations:** ^1^Department of Neuroscience, Johns Hopkins University School of MedicineBaltimore, MD, USA; ^2^Department of Biochemistry and Molecular Biology, University of British ColumbiaVancouver, BC, Canada

**Keywords:** NMDA receptor, neurotrophins, Trk receptors, signaling endosomes, endocytic recycling, neurites, dendritic spines, protons

## Abstract

Neuronal precursor cells extend multiple neurites during development, one of which extends to form an axon whereas others develop into dendrites. Chemical stimulation of N-methyl D-aspartate (NMDA) receptor in fully-differentiated neurons induces projection of dendritic spines, small spikes protruding from dendrites, thereby establishing another layer of polarity within the dendrite. Neuron-enriched Na^+^/H^+^ exchanger NHE5 contributes to both neurite growth and dendritic spine formation. In resting neurons and neuro-endocrine cells, neuron-enriched NHE5 is predominantly associated with recycling endosomes where it colocalizes with nerve growth factor (NGF) receptor TrkA. NHE5 potently acidifies the lumen of TrkA-positive recycling endosomes and regulates cell-surface targeting of TrkA, whereas chemical stimulation of NMDA receptors rapidly recruits NHE5 to dendritic spines, alkalinizes dendrites and down-regulates the dendritic spine formation. Possible roles of NHE5 in neuronal signaling via proton movement in subcellular compartments are discussed.

## Introduction

A robust fluctuation in pH_*i*_ (pH *i*nside of the cell, or cytosolic pH) and pH_*o*_ (pH *o*utside of the cell or extracellular pH) greatly influence synaptic transmission, which is often associated with pathological conditions (Velisek, 1998; Hsu et al., 2000; Chesler, 2003). For example, seizure leads to interstitial acidification of hippocampal neurons (Somjen, 1984) and excessive acidosis inhibits synaptic transmission and eventually causes coma (Li and Siesjö, 1997), while alkalinization may induce seizures (Schuchmann et al., 2006). Conversely, strong synaptic stimulation initiates a series of changes in pH_*o*_ in the vicinity of the synapse beginning with an immediate acidification lasting a few milliseconds followed by a slower alkaline transient for several 100 ms (Chesler and Kaila, 1992). Epileptiform activity induced by low-Mg^2+^ was suppressed by acidic media (Velisek et al., 1994). While global changes in pH_*i*_ and pH_*o*_ may cause perilous effects on neuronal functions, transient (e.g., during neuronal development, in response to neuronal stimuli) and localized changes in pH in subcellular compartments (e.g., neuronal synapses and endosomal lumens) occur under non-pathological states and regulate neurological function.

Most of glutamate receptors including N-methyl-D-aspartate (NMDA) receptors, α-amino-3-hydroxy-5-methyl-4-isoxazolepropionic acid (AMPA) receptors (Ihle and Patneau, 2000), Kainate receptors (Mott et al., 2003), and the group III metabotropic glutamate receptors (Levinthal et al., 2009) are down-regulated by extracellular protons. Among them, NMDA receptors are particularly sensitive to extracellular protons because of the physiological pK_*a*_ of 7.3–7.5 (Tang et al., 1990; Traynelis and Cull-Candy, 1990; Banke et al., 2005). Given the importance of NMDA receptors for synaptic plasticity, this suggests that local pH in the vicinity of the synapse may be important for the induction and maintenance of *l*ong-*t*erm *p*otentiation (LTP) and *l*ong-*t*erm *d*epression (LTD). Consistent with this notion, high-frequency stimulation of axon projections from the hippocampus CA3 region, termed Schaffer collaterals, failed to induce LTP in hippocampal CA1 pyramidal neurons when the cells were bathed in an acidified media, likely a result of NMDA receptor inhibition (Velisek, 1998). pH_*o*_ changes at the confined space of synaptic cleft may coordinate synaptic excitability by synergistically regulating excitatory and inhibitory receptors of post-synaptic neurons in a reciprocal manner. The best-studied pH_*o*_-sensitive ion translocating mechanism would be *A*cid *S*ensing *I*on *C*hannels (ASICs), proton-gated ion channels predominantly expressed in the nervous system, which are involved in pain, seizure, stroke and anxiety-related neurological disorders (Wemmie et al., 2008; Gründer and Chen, 2010; Zha, 2013). Acidic pH_*o*_ facilitates the chloride conductance of certain *g*amma *a*mino *b*utyric *a*cid (GABA) receptors, which inhibits firing of action potentials (Krishek et al., 1996; Dietrich and Morad, 2010). It has been also suggested that the pre-synaptic K^+^-current is regulated by pH_*o*_ (Almanza et al., 2008).

Endosomes are membrane-bound orgaenelles that internalize membrane components and external molecules. Internalized vesicles are delivered to lysosomes for degradation or return to the plasma membrane via endocytic recycling pathways (Maxfield and Mcgraw, 2004). The endosomal lumen is acidic (Casey et al., 2010; Scott and Gruenberg, 2011), which is established by Vacuolar proton-translocating ATPases (V-ATPases) and the counter-ion conductance by anion channels/transporters such as the Cl^−^ channel and Cl^−^/H^+^ exchanger ClC family (Forgac, 2007; Stauber and Jentsch, 2013). Anion channels/transporters relieve the charge-imbalance by pumping anions into the lumen and facilitate continuous action of V-ATPases to pump protons to the lumen. In neurons and neuroendocrine cells, the neuron-enriched Na^+^/H^+^ exchanger NHE5 seems to play an equally important role as V-ATPases. A unique aspect of NHE5 is that it acts in both endosomes and synapses. We discuss the potential role of proton concentration oscillations by NHE5 in neuronal signaling.

## Na^+^/H^+^ exchangers in neurons

*N*a^+^/*H*^+^ *e*xchangers (NHEs) are a group of secondary active antiporters that typically exchange extracellular Na^+^ for cytosolic protons in a 1:1 ratio, thereby regulating cellular pH and cell volume (Aronson, 1985; Wakabayashi et al., 1997; Orlowski and Grinstein, 2004; Slepkov et al., 2007). The recovery from acute acidification in biochemically-isolated synapses requires external Na^+^ whereas HCO^−^_3_ deprivation or inhibitors against bicarbonate transporters also affect pH_*i*_ of some neurons (Sauvaigo et al., 1984; Nachshen and Drapeau, 1988; Chesler, 2003). Although Na^+^-driven Cl^−^-HCO^−^_3_ exchange activity was detected in freshly dissociated hippocampal neurons (Schwiening and Boron, 1994) and some neuronal cell populations may require bicarbonate-dependent pH regulation mechanisms, Na^+^-dependent recovery from acute cytosolic acidification occurs even in bicarbonate-free media (Raley-Susman et al., 1991). Thus, NHEs play crucial roles in pH regulation in neurons while HCO^−^_3_ is also likely an important pH regulator of certain types of neurons. NHE-activity indeed regulates synaptic transmission at glutamatergic, GABAergic and dopaminergic synapses (Trudeau et al., 1999; Jang et al., 2006; Rocha et al., 2008; Dietrich and Morad, 2010) and there is evidence suggesting that local pH contributes to the induction and maintenance of LTP (Velisek, 1998; Ronicke et al., 2009; Diering et al., 2011). In mammals, nine NHE isoforms NHE1-NHE9 have been characterized as secondary active ion transporters (Brett et al., 2005; Donowitz et al., 2013). NHE1-NHE5 are functionally well-defined NHEs that exhibit the typical Na^+^-H^+^ exchange activity, of which ubiquitously expressed NHE1 and neuron-enriched NHE5 are the two predominant isoforms in the brain (Attaphitaya et al., 1999; Baird et al., 1999). NHE6-NHE9 exhibit “atypical” *c*ation *n*on-specific *o*rganellar activity across acidic organellar membranes (Orlowski and Grinstein, 2007; Ohgaki et al., 2011) therefore referred to as CNO-NHEs. Since CNO-NHEs have a higher affinity to K^+^ than Na^+^, their physiological mode of action is likely to “leak” protons from the lumen of acidic organelles driven by the influx of K^+^, the major cytosolic monovalent cation. The unique cation-nonspecific antiporter activity was reported first in NHE7 in a heterologous expression system (Numata and Orlowski, 2001) and a similar activity was subsequently detected in other CNO-NHEs in an *in vitro* reconstitution system (Nakamura et al., 2005). More recent studies showed that genetic depletion of NHE6 leads to endosomal acidification (Ohgaki et al., 2010; Xinhan et al., 2011; Ouyang et al., 2013) in neuronal and non-neuronal cells, supporting the physiological relevance of CNO-NHEs for proton-leak from acidic organelles. Although widely expressed in most mammalian cell types, genetic alterations in NHE6, NHE7, and NHE9 have been associated with X-linked mental retardation syndrome (Gilfillan et al., 2008; Schroer et al., 2010; Takahashi et al., 2011; Mignot et al., 2013), late-onset Alzheimer's disease (Meda et al., 2012), and autism spectrum disorders and attention-deficit/hyperactivity spectrum disorder (ADHD) (Lasky-Su et al., 2008; Morrow et al., 2008; Markunas et al., 2010; Mick et al., 2010), respectively.

NHE1 null-mice exhibit neurological phenotypes including epileptic-like seizures resulting from enhanced neuronal excitability and loss of Purkinje cells in cerebellum (Cox et al., 1997; Liu et al., 2013), but otherwise brain development occurs without major complications. This suggests that while selected neurons such as Purkinje cells are particularly sensitive to pH changes, other neurons possess distinct mechanisms to defend against a pH challenge. Unique NHE-activity distinct from NHE1 has been detected in hippocampal neurons, which is relatively insensitive to amiloride, highly sensitive to ATP-depletion and has a high affinity to Li^+^ (Raley-Susman et al., 1991; Schwiening and Boron, 1994; Baxter and Church, 1996). NHE5 is ~100-fold more resistant to amiloride than NHE1 (Masereel et al., 2003) and has a higher affinity to Li^+^ than NHE1 (Szabo et al., 2000). Moreover, ATP-depletion almost completely abolishes NHE5 activity (Szabo et al., 2000) whereas NHE1 is only partially suppressed by ATP-depletion (Kapus et al., 1994). Thus, NHE5 is most likely the responsible molecule for non-NHE1 type NHE activity physiologically detected in hippocampal neurons. The highly ATP-sensitive nature implies a possible role of NHE5 in pathological processes such as ischemia and reperfusion. While NHE5 is predominantly associated with recycling endosomes and potently acidifies the lumen in resting neuroendocrine model cells (Diering et al., 2013), it is acutely targeted to dendritic spines upon neuronal activation [(Diering et al., 2011) and see below]. Movement of protons from the cytosol to the endosomal lumen via NHE5 should theoretically affect the global cytosolic pH (pH_*i*_), however this depends on a number of other factors including the volume of recycling endosomes and the buffering power, and the actual effect of NHE5 on the overall cytosolic pH is not clear. Currently NHE5 knockout mice are not available. In summary, currently available data suggest that ubiquitous Na^+^/H^+^ exchanger NHE1, neuron-enriched NHE5 and CNO-NHEs across organellar membranes are the three major NHEs in neurons.

## Local pH modulates dendritic spine morphology

Most forms of LTP require activation of synaptic NMDA receptors (NMDARs) (Malenka and Bear, 2004) and subsequent calcium influx. This initiates a signaling program that eventually recruits AMPA receptor to synapses (Shepherd and Huganir, 2007) and facilitates the formation of dendritic spines (Engert and Bonhoeffer, 1999; Maletic-Savatic et al., 1999; Lang et al., 2004; Matsuzaki et al., 2004). NMDARs have especially high proton sensitivity with a pK_*a*_ of 7.3–7.5 (Tang et al., 1990; Traynelis and Cull-Candy, 1990) due to a discrete extracellular proton binding site distinct from other ligand binding sites (Banke et al., 2005). The proton binding site is associated with the channel gating mechanism such that channel open probability is strongly suppressed by proton binding. Unlike the voltage-dependent block of NMDARs by Mg^2+^ ions (Nowak et al., 1984; Kumamoto, 1996), this proton block is not dependent on voltage. Thus, at resting extracellular pH close to 7.3, a tonic proton block exists, which maintains NMDAR-activity to about 50%, which means that any slight deviation in local pH should theoretically have a profound impact on synaptic transmission and synaptic plasticity. Therefore, synaptic pH-regulating ion transporters like NHE5 may contribute to a pH-based inhibitory tone to limit synaptic transmission and synaptic plasticity.

**Figure 1:**
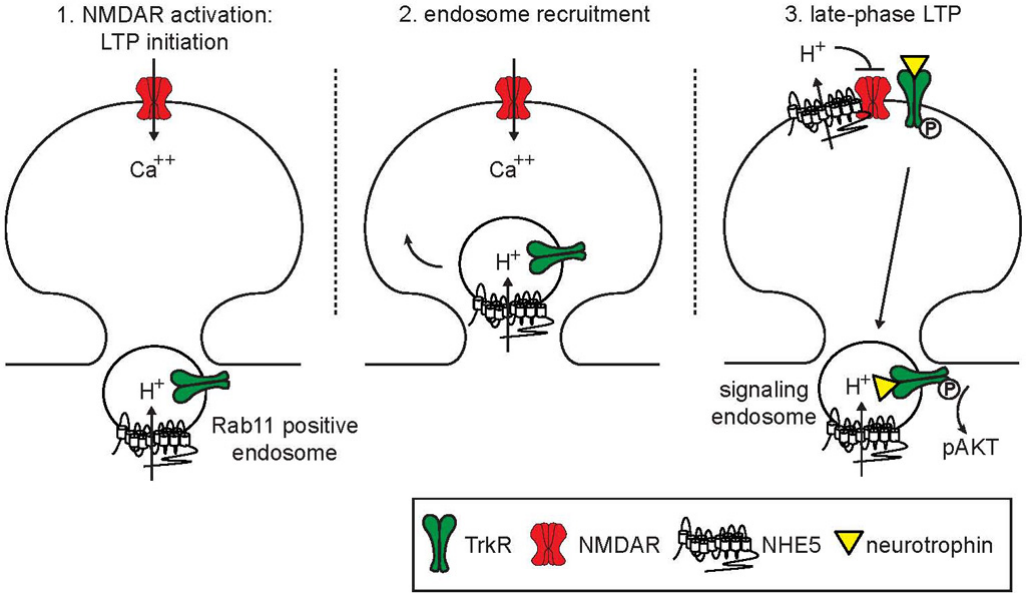
**Model of endosomal and synaptic functions of NHE5**. At steady state, NHE5 is primarily associated with recycling endosomes, where it acts to acidify the lumen of this compartment. Upon activation of NMDA receptors, recycling endosomes containing NHE5 and Trk receptors are mobilized and recruited to active synapses. One possibility is that signaling downstream of NMDA receptors acutely stimulates NHE5 activity in the endosomal membrane, driving endosomal acidification and promoting endosomal recycling. Following insertion, NHE5 is active on plasma membrane, acting to supress further NMDA receptor activity through localized acidification of the synaptic cleft. In addition, active NHE5 can support the surface expression of Trk receptors and enhance AKT signaling from recycling endosomes. These coordinated activities may help the synapse transition from an initiation phase of LTP into a consolidation phase, limiting excitotoxicity from sustained NMDA receptor activation while promoting synapse strengthening through local neurotrophin signaling.

Under basal conditions, NHE5 is predominantly localized to recycling endosomes. Within minutes following NMDAR activation by co-agonist glycine, NHE5 is recruited into dendritic spines and exposed onto the cell surface at excitatory synapses. Stimulation of NMDARs by glycine is often referred to as “chemical LTP” because this manipulation induces long-lasting mEPSC, which is mediated by AMPA receptor components (mEPSC_AMPA_) and is inhibited by NMDAR inhibitors (Lu et al., 2001). Dendritic spines then undergo an NHE5-dependent alkaline shift in their pH_*i*_ as NHE5 transports cytosolic protons into the extracellular space (Diering et al., 2011). A model is proposed in which NHE5 targeted to dendritic spines by NMDAR-activation acutely mobilizes protons across the post-synaptic membrane, which in turn down-regulates NMDAR, thereby forming a negative-feedback loop (Figure 1). As neurological disorders such as depression, schizophrenia and autism are associated with aberrant NMDAR activity and dendritic spine morphogenesis (Lakhan et al., 2013; Zhou and Sheng, 2013), it is tempting to speculate that NHE5 may be involved in pathogenic processes of these diseases. Recently, Deane and colleagues reported that chemical LTP enhances translocation of NHE6 to dendrites in mouse hippocampal neurons (Deane et al., 2013), resembling the targeting behavior of NHE5 in rat hippocampal neurons (Diering et al., 2011). Small populations of CNO-NHEs are indeed targeted to the plasma membrane in non-neuronal cells (Lin et al., 2007; Kagami et al., 2008; Ohgaki et al., 2008; Onishi et al., 2012) and in vestibular hair cells (Hill et al., 2006); however, whether CNO-NHEs transiently delivered to the plasma membrane exhibit (Na^+^, K^+^)/H^+^ exchange activity remains to be determined. Another important unanswered question is whether NHE6 participates in activity-dependent pH regulation of dendritic spines. Future investigations are needed to address these important mechanistic questions. Curiously, acid-sensing ion channel ASIC1a is present in dendritic spines, serves as a pH-sensor and influences the density of spines (Zha et al., 2006), raising an interesting possibility that ASIC1 in dendritic spines may be regulated by NHE5 and possibly NHE6.

## Endosomal acidity in Trk targeting and signaling

Binding of neurotrophins [e.g., nerve growth factor (NGF), brain-derived neurotrophic factor (BDNF) and neurotrophin-3] to their high affinity TrkA, TrkB and TrkC stimulates downstream signaling, leading to neuronal differentiation and survival (Huang and Reichardt, 2001; Chao, 2003). Vectorial targeting of the NGF-receptor TrkA from the endosomal pool to growing neurite tips serves as a regulatory mechanism for neurotrophin signaling (Arimura et al., 2009; Vaegter et al., 2011) and neurite outgrowth (Ascano et al., 2009). Similarly, endocytic recycling recruits TrkB to the post-synaptic density during LTP that is required for dendritic branching (Huang et al., 2013; Lazo et al., 2013). Thus, endocytic recycling not only regulates the cell-surface availability of Trk proteins but also provides the accurate targeting path to neurites.

Rat pheochromocytoma PC12 cells, widely used as a neuroendocrine model (Greene and Tischler, 1976), have more acidic recycling endosomal pH of ~6.2 (Diering et al., 2013) than recycling endosomal pH of ~6.5 in fibroblasts (Maxfield and Mcgraw, 2004; Scott and Gruenberg, 2011). NHE5-depletion by shRNA or V-ATPase-inhibition by Bafilomycin lead to a similar degree of alkalinization of recycling endosomes to pH = ~6.5 in PC12 cells, whereas concomitant inhibition of NHE5 and V-ATPase further alkalinizes recycling endosomes to pH = ~6.75. These results indicate that NHE5 acidifies recycling endosomes as potently as V-ATPases in PC12 cells. NHE5 and TrkA colocalize intracellularly by double immunofluorescence microscopy and NHE5-depletion reduces cell-surface targeting of TrkA, and impairs NGF-induced neurite formation; while V-ATPase inhibitor Bafilomycin has also decreased the cell-surface population of TrkA (Diering et al., 2013). Importantly, NHE5 depletion as well as V-ATPase inhibition has impaired endocytic recycling of TrkA but not transferrin receptor (TfnR) or Na^+^/K^+^-ATPase. These results suggest that endosomal acidification is important in the polarized targeting of specific endosomal cargoes in PC12 cells. It was recently reported that NHE6 deletion impairs neurotrophin signaling and affects axonal and dendritic branching of hippocampal neurons (Ouyang et al., 2013). Genetic depletion leads to excessive acidification of late endo-lysosomes and facilitates degradation of TrkB, which in turn decreases responsiveness to BDNF. However, a significant population of NHE6 seems to be associated with recycling endosomes and other yet-to-be identified intracellular compartments (Ouyang et al., 2013). NHE6 depletion was shown to acidify recycling endosomal pH in HeLa cells (Xinhan et al., 2011) and hepatocytes (Ohgaki et al., 2010), and NHE6 in hepatocytes affects polarized distribution of membrane lipids to the apical surface. Taken together, it is possible that NHE6 in neurons regulates endocytic recycling of TrkB. We suggest that the balanced action of NHE5, NHE6 and perhaps other CNO-NHEs in different organelles along the endocytic and recycling pathway is needed for proper targeting of Trk proteins, and impairment of any of their function may affect neurotrophin signaling.

Although both phosphatidylinositol 3-kinase (PI3K)-Akt and extracellular signal-regulated kinase (Erk) signaling pathways are downstream of NGF-TrkA, PI3K-Akt signaling seems to be more severely affected by luminal alkalinization, suggesting that endosomal pH may directly influence PI3K-Akt signaling in addition to its role in TrkA targeting. Indeed, Akt signaling occurs in endosomes in various cell types (Garcia-Regalado et al., 2008; Schenck et al., 2008; Walz et al., 2010; Nazarewicz et al., 2011) including NGF-treated PC12 cells (Lin et al., 2006; Varsano et al., 2006). Endosomes provide a confined space that allows for continuous signaling from the internalized ligand-receptor complex (Gould and Lippincott-Schwartz, 2009; Murphy et al., 2009; Platta and Stenmark, 2011) and certain signaling may arise in endosomes (Sorkin and von Zastrow, 2009; Scita and Di Fiore, 2010). Thus, although the plasma membrane is undoubtedly the most important cellular location for the initial activation of PI3K-Akt signaling, sustained signaling does occur in endosomes where pH may modulate the signaling intensity.

Trk neurotrophin receptors are likely associated with diverse endosomal populations. Aside from the recycling endosomal (Diering et al., 2013) and late endosomal pH (Ouyang et al., 2013), early endosomal pH has been suggested to influence neurotrophin signaling by modulating the neurotrophin-receptor binding and cell-surface targeting that is mediated by a small GTPase Rac1 and coffilins (Harrington et al., 2011). As such, it is possible that non-selective perturbation of all the acidic compartments by Bafilomycin or weak alkaline agents may lead to mixed biological effects. In future studies, it will be important to dissect the precise role of pH in different organellar compartments in neurotrophin signaling.

## Concluding remarks

Compelling experimental evidence suggests that synaptic functions are tightly controlled by endocytic recycling (Park et al., 2006; Wang et al., 2008). While the importance of pH in localized electrical activity, cell signaling and vesicular trafficking is well recognized, the molecular basis underlying compartmentalized pH regulation in neurons has been lacking. We now suggest that the recycling plasma membrane type NHE5 in neuroendocrine and neuronal cells potently acidifies recycling endosomes and modulates signaling events occurring in endosomes and synapses. Though still in its early stage, NHE5 offers an example of how localized pH regulation can impact synaptic plasticity and neuronal differentiation.

### Conflict of interest statement

The authors declare that the research was conducted in the absence of any commercial or financial relationships that could be construed as a potential conflict of interest.
